# Information storage in permalloy modulated magnetic nanowires

**DOI:** 10.1038/s41598-021-00165-1

**Published:** 2021-10-21

**Authors:** Guidobeth Sáez, Pablo Díaz, Eduardo Cisternas, Eugenio E. Vogel, Juan Escrig

**Affiliations:** 1 Department of Physics, Universidad de La Frontera, Casilla 54-D, Temuco, Chile; 2 Center of Nanoscience and Nanotechnology (CEDENNA), 9170124 Santiago, Chile; 3 Departamento de Física, Universidad de Santiago de Chile (USACH), Avda. Ecuador 3493, 9170124 Santiago, Chile

**Keywords:** Materials science, Condensed-matter physics, Materials for devices, Nanoscale materials

## Abstract

A long piece of magnetic material shaped as a central cylindrical wire (diameter $d=50$ nm) with two wider coaxial cylindrical portions (diameter $D=90$ nm and thickness $t=100$ nm) defines a bimodulated nanowire. Micromagnetism is invoked to study the equilibrium energy of the system under the variations of the positions of the modulations along the wire. The system can be thought of as composed of five independent elements (3 segments and 2 modulations) leading to $2^5=32$ possible different magnetic configurations, which will be later simplified to 4. We investigate the stability of the configurations depending on the positions of the modulations. The relative chirality of the modulations has negligible contributions to the energy and they have no effect on the stability of the stored configuration. However, the modulations are extremely important in pinning the domain walls that lead to consider each segment as independent from the rest. A phase diagram reporting the stability of the inscribed magnetic configurations is produced. The stability of the system was then tested under the action of external magnetic fields and it was found that more than 50 mT are necessary to alter the inscribed information. The main purpose of this paper is to find whether a prototype like this can be complemented to be used as a magnetic key or to store information in the form of firmware. Present results indicate that both possibilities are feasible.

## Introduction

Nanometric materials are not only smaller than their macro counterparts but they also exhibit new properties^1,2^. Among the different nanometric structures, nanowires are the focus of different research groups due to their high aspect ratio, physicochemical properties, together with outstanding mechanical, electrical, magnetic and optical properties, which can also be controlled by varying their geometrical parameters. Furthermore, nanowires are used in many technological applications in different fields like nanoelectronics^3,4^, magneto-optoelectronics^5–8^, magneto-plasmonics^9^ and even in wearable electronic systems^10^, among others.

Magnetic nanowires with a square cross-section are known as planar nanowires and are obtained mainly by lithographic techniques. However, magnetic nanowires with circular cross-section are the ones that concentrate most of the interest today due to their curved surface that may cause the curvature-induced effective anisotropy or chiral symmetry breaking^11^, being used in potential applications^12–14^. Furthermore, cylindrical nanowires favor the formation of unconventional magnetic textures whose dynamics differ considerably from those that appear in two-dimensional wires^15^. In fact, in cylindrical systems, Walker breakdown occurs at very high current densities^16^ or can even be suppressed^17^; thus, once domain walls are depinned, they are expected to move at very high velocities.

In order to synthesize cylindrical nanowires there are mainly two ways: template-free^18,19^ or template-assisted^20–22^ methods. This last method based on self-organized porous membranes has made notable advances, allowing us to dream of the design proposed by Parkin in 2008 of a racetrack memory^23^. This memory is based on the fact that information can be stored in a solid-state device through magnetic domains (areas where magnetic moments point in a defined direction) separated by magnetic domain walls (areas where magnetic moments vary from the direction of one domain to the other). These domain walls can be moved either by applying an external magnetic field^20,24,25^, a spin-polarized current^17^, spin waves^26^ or localized temperature gradients^27^. The idea is that the position of these magnetic domain walls can be precisely controlled through pinning centers, which can be generated by varying the composition of the nanowire^28–30^ or by introducing geometrical inhomogeneities^20,24,31–39^, such as modulations in its diameter during the synthesis process. Diameter modulations of the nanowire effectively allow controlling the domain wall positions since they locally reduce the magnetostatic and exchange energy in the different cross-sectional parts^40–48^. Recently, Salem et al.^49^ went further and succeeded in synthesizing magnetic nanowire arrays by modulating both their composition and their diameters using a new synthesis method. Although we propose here permalloy as the material of study, this is merely by simplicity, bearing in mind that the search for properties in nanowires of other materials is a very actual research field^50^.

In this article, we assume that diameter modulated nanowires can eventually store firmware. Thus, one of the main goals of the present paper points to recognize the main role of the modulations: Are they simple spacers or could they also be used to store information? Then, we want to study the stability of the inscribed magnetic orientations along the segments (thinner elements between modulations): what are the conditions for a stable inscribed magnetic configuration that does not spontaneously reverse the magnetization in some segments? The robustness of the system will then be tested with respect to externally applied magnetic fields (accidental or intentional). When solving this problem, we will also find some other interesting fundamental features like the presence of Bloch Points (BPs) in some of the configurations. BPs are not an objective of the present paper, but we will just unfold them since they are an intrinsic property of the system proposed here.

Our system then consists of a solid and homogeneous cylindrical piece of magnetic material modulated in diameter along the axis. We assume that modulations are wider in diameter than the central part that is then divided into segments: there are *N* modulations and ($N+1$) segments all concentric along the axis. The parameter space includes at least the following geometrical properties: length *L* of the total nanowire, number of modulations *N*, thickness of the individual modulations $t_i$ ($i=1,2,..N$), diameter of the individual modulations $D_i$ ($i=1,2,..N$), length of the individual segments $\ell _j$ ($j=1,2,..N+1$), diameter of the individual segments $d_j$ ($j=1,2,..N+1$), with only one constraint:

1 $$\begin{aligned} \sum _{i=1}^{N}t_i \, +\, \sum _{j=1}^{N+1} \ell _j=L. \end{aligned}$$

Such complex parameter space necessarily needs to be filtered to focus on the main general properties of the System: the possibility to inscribe a desired magnetic configuration that will be robust enough so it does not reverse spontaneously and it is also stable even when weak or moderate external magnetic fields are applied to it. This task will be done in the next section.

This paper is organized in the following way: the next section describes the System and the methodology; the third section is devoted to Results and discussions, followed by the section with Conclusions. Additionally, we prepared a separate file with Supporting Information to provide for a wider basis for deeper discussions.

## System and methodology

### System

**Figure 1:**
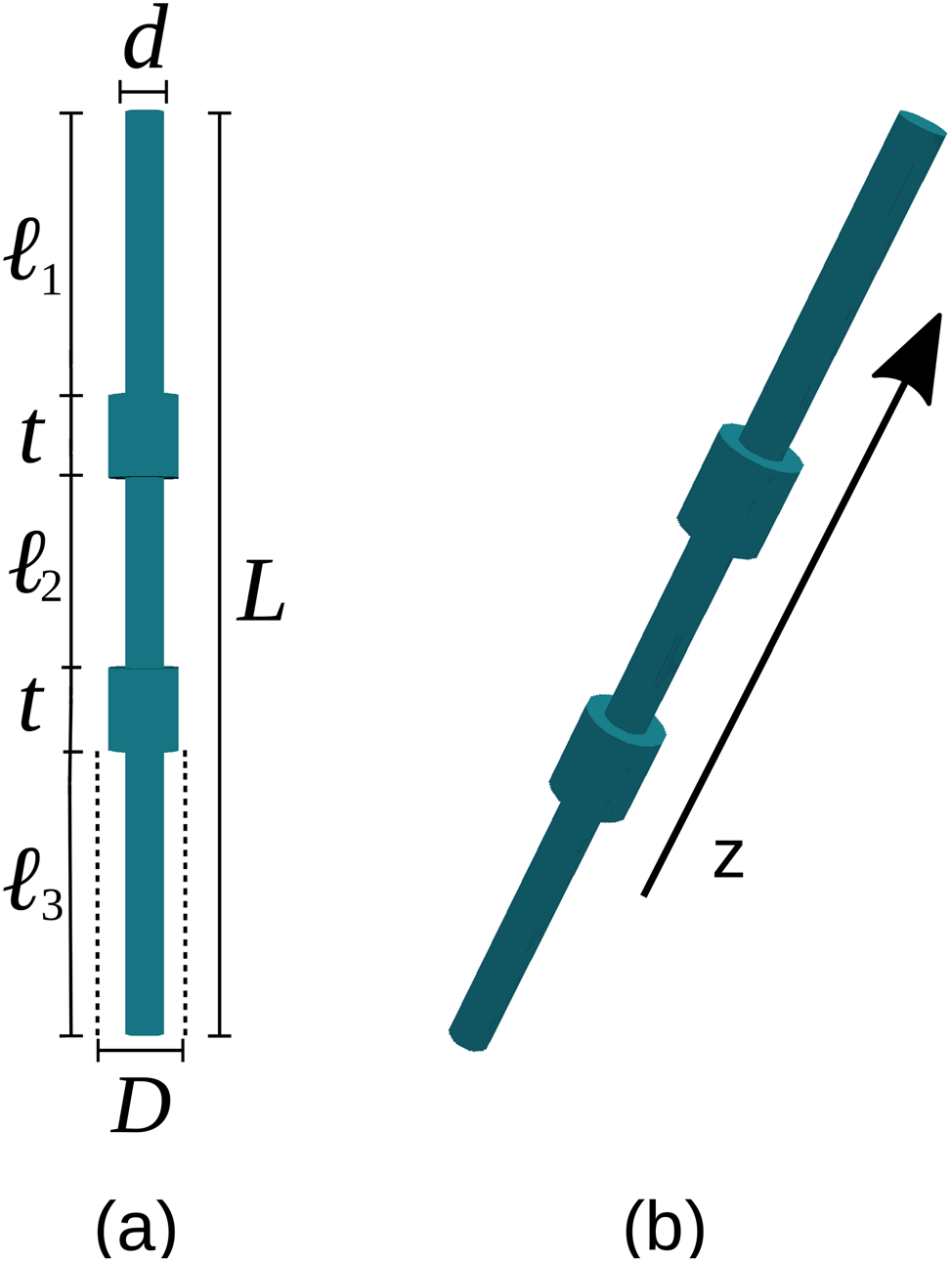
**(a)** Front view of the proposed bimodulated nanowire with the definition of its geometrical parameters. **(b)** 3D view of the system where its symmetry axis is parallel to the z-axis along which a magnetic field will be later applied.

Let us consider an isolated permalloy nanowire as illustrated in Fig. 1. We perform our calculations for a device with a representative length $L=1100$ nm with two modulations $\mu _i$ ($i=1,2$) in diameter along its axis, which divide the main nanowire into three segments $S_j$ ($j=1,2,3$). To further simplify the parameter space, we make all thicknesses ($t_i$) and diameters ($D_i$, $d_j$) the same. Namely, $t_i=t= 100$ nm; $D_i=D= 90$ nm for all *i*; and $d_j=d=50$ nm for all *j*. Thus, the only free parameters left are $\ell _1$, $\ell _2$, and $\ell _3$, namely, the lengths of the segments limited by the modulations with the constraint $\ell _1+\ell _2+\ell _3=900$ nm. This choice of parameters was motivated by a previous result showing that the coercivity of a monomodulated magnetic nanowire depended strongly on its position along the axis^51^.

In general, the aspect ratio between the length and diameter of a ferromagnetic cylinder defines three possible minimum energy configurations: ferromagnetic in-plane (I), ferromagnetic out-of-plane (II) and vortex (III)^52^. If segments are long enough their preferred magnetization will point in any of the two directions along the axis^53^. By a similar token, *D* and *t* can be chosen so the dominant magnetization within the modulation (bare disk) is a vortex, where chirality can be clockwise or counterclockwise. To focus on how the geometrical parameters of the system influence its magnetic properties, we consider permalloy nanowires, since this material does not exhibit noticeable crystalline anisotropy^54^ like Co^55^ or Ni^50^ for example.

Although the previous set of parameters is a bit arbitrary, it represents the kind of system we are looking for: a simple device with magnetically separate elements capable of storing stable enough magnetic configurations. Variations to these values could modulate the behavior reported below, but the general properties of the device are already established.

### Magnetic configurations

The entire system is composed of 5 elements: three segments and two modulations. This realization should be considered as a first prototype only; from here, several variations in the number of modulations and variations of geometrical parameters are possible. Due to shape anisotropy, magnetization in the segments is largely axial with two possible orientations. Similarly, magnetization in the isolated modulations tends to be in the vortex phase with two chirality twists. Altogether there are $2^5=32$ independent possible configurations. The Fig. 1 in the Supporting Information illustrates each one of these configurations. Due to the symmetry in the Hamiltonian they are doubly degenerate, so we need to study the energy of only 16 of them; the other 16 have the same energy as those configurations reached upon reversing every single spin. Moreover, energy difference due to the different relative chiralities in the modulations turns out to be of the order of 0.01 eV as presented in Fig. 4 of the Supporting Information. Such energy difference is negligible compared to the other energies under consideration here, like the energy difference observed when two contiguous segments have opposite magnetization, which turns out to be of the order of 100 eV. The inscribed configurations are allowed to evolve according to the dynamics presented in the next Subsection. In this way, the arbitrary domains imposed at the beginning of the simulations evolve to more relaxed magnetic configurations looking for better defined metastable magnetic states. This is shown in Fig. 2 of the SI for configurations $G_9$, $G_{13}$ and $G_{14}$: the top panel corresponds to the initial configuration; the lower panel illustrates the field distribution way after reaching equilibrium (10 ns). When segments of opposite polarity meet inside the modulations, conditions propitious to host Bloch Points arise as depicted by the cones present at the corresponding interfaces.

**Figure 2:**
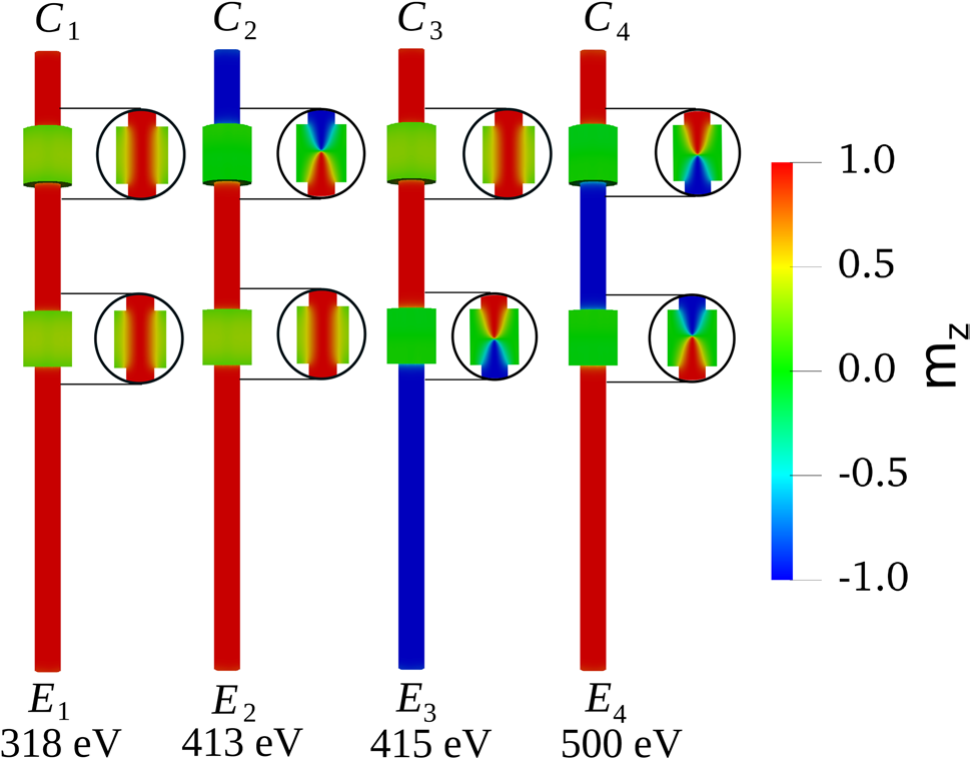
The final 4 energetically distinguishable magnetic configurations in order of increasing equilibrium energy. Magnetization values are approximately illustrated by a color code defined to the right. The magnetization distributions inside the modulations are shown by circled cuts to the right. When modulations host end of segments with different magnetic orientations, Bloch points naturally appear, and they can be recognized by their conical shape: $C_2$ and $C_3$ present one BP each, while $C_4$ exhibits a BP inside each of the two modulations.

Moreover, such equilibrium energy differences lose significance even when compared to the thermal noise present at normal room temperatures. Thus, we need to consider only 4 independent and energetically different configurations coming from the magnetic orientations along the segments. These four configurations are shown in Fig. 2 for $\ell _1=135$ nm, $\ell _2=225$ nm and $\ell _3=540$ nm as an example. $C_1$ with the magnetization of the three segments pointing in the same direction (true ground configuration); $C_2$ with the magnetization of the middle segment along the magnetization of the longest external segment; $C_3$ with the magnetization of the middle segment along the magnetization of the shortest external segment; $C_4$ with the magnetization of the middle segment opposite to the magnetization of both external segments.

These configurations could be inscribed in the system in different ways. As an example, let us consider configuration $C_4$ of Fig. 2 to illustrate a possible way of achieving this purpose. Let us expose the entire device to an external strong magnetic field that will orientate all the three segments in the blue direction. Then we approach to the upper end a magnetic field strong enough to reverse the magnetization of that end only. Finally, we do a similar job on the other end reversing that portion from blue to red. Similarly, configurations $C_2$ and $C_3$ can be inscribed. Any of these combinations, plus the different lengths of the segments, can be used to store information in the form of firmware or a magnetic key. The purpose of the rest of the paper is precisely to test the stability of the inscribed information depending on the geometry of the device.

### Geometrical parameter space

The parameter space has only two free variables since the sum of the segments is limited to 900 nm. Let us separate $\ell _2$ as a singular variable due to its different role. Then the other two segments can be better considered by their relative lengths in the following way: we define $\zeta = 1 - \frac{\ell _1}{\ell _3}$ for $\ell _1 < \ell _3$ (positive values) and as $\zeta = \frac{\ell _3}{\ell _1} -1$ for $\ell _3 < \ell _1$ (negative values). If $\zeta =0$ the segments at the ends have equal length, regardless of the value of $\ell _2$; while if $\zeta =+1(-1)$ it implies that the upper (lower) segment does not exist. However, we set a minimum value of 50 nm for either $\ell _1$ or $\ell _3$ to preserve the axial symmetry in these segments.

**Figure 3:**
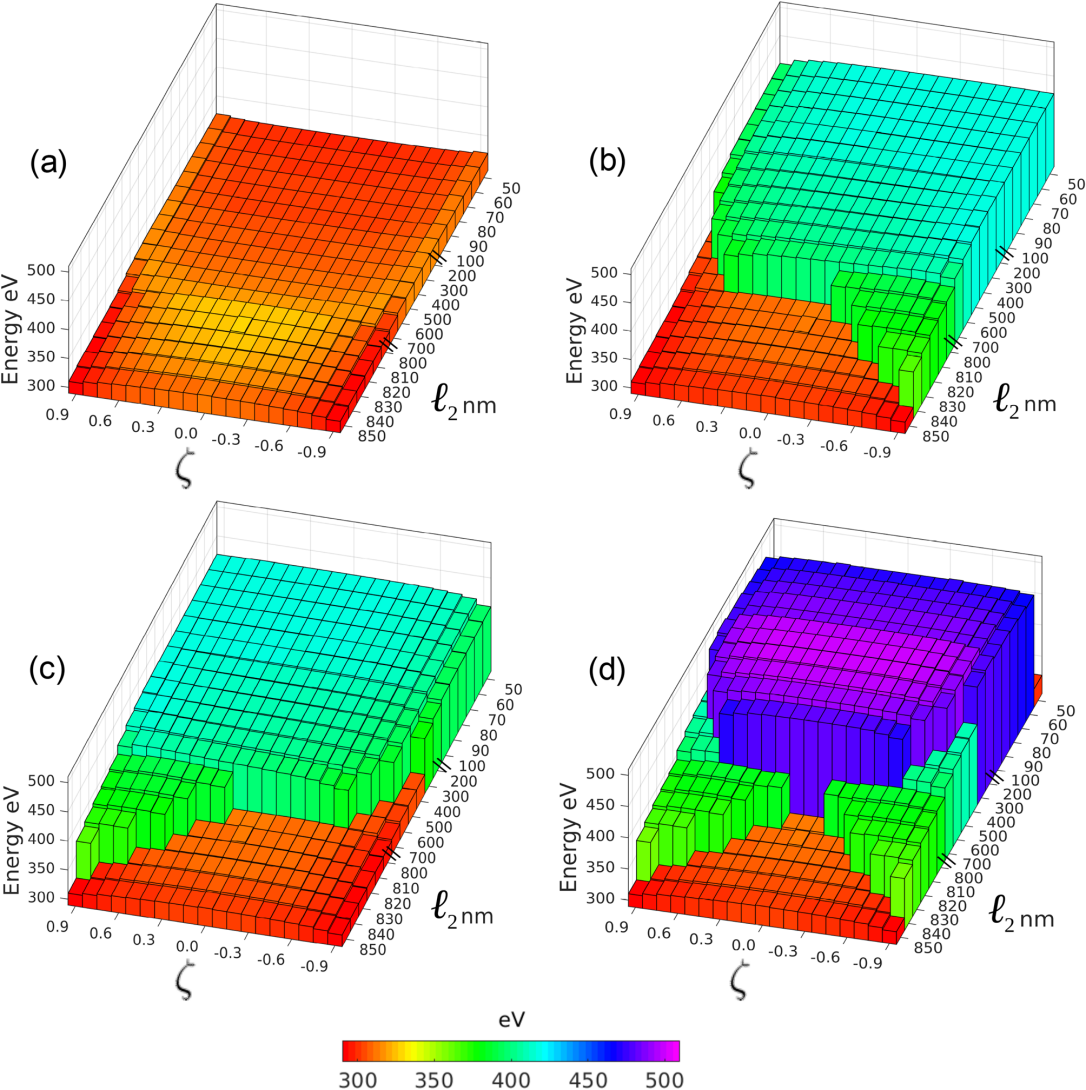
Energy phase diagram testing stability of the inscribed information. Each pixel represents a different $\ell _1$, $\ell _2$ and $\ell _3$ (through $\zeta$) choice and the height gives the corresponding equilibrium or final energy. Panel **(a)** illustrates the equilibrium energy for an original configuration $C_1$ inscribed in the system: obviously here it remains in the true ground $C_1$ configuration although the final energy landscape is not flat. Panel **(b)** corresponds to a $C_2$ configuration initially inscribed in the system represented by light gray (blue/green) color; however, for some geometries this initial configuration is unstable and decays to $C_1$ represented by the corresponding gray (orange) color and lower energies. Panel **(c)**, with $C_3$ originally inscribed, is symmetric to panel **(b)** with respect to the sign of $\zeta$. Panel **(d)** represents systems initiated as $C_4$ configuration; those remaining as $C_4$ are marked in dark gray (blue/violet), but some of them are not stable and spontaneously decay to $C_2$ or $C_3$ for large enough $\ell _2$ values and not very small $|\zeta |$ as represented by light gray color (blue/green); configurations with both large values of $\ell _2$ and small $|\zeta |$ can collapse directly into $C_1$ configurations as presented by pixels in gray (orange) color.

For this two-dimensional parameter space, $\{\ell _2,\zeta \}$, we consider that $\ell _2$ takes values between 50 nm and 850 nm and $\zeta$ takes values between $+0.9$ and $-0.9$. To optimize the computational time we have considered different steps for $\ell _2$ in three ranges: $\ell _2 = 50 - 100$ nm with $\Delta \ell _2 = 10$ nm, $\ell _2=100-800$ nm with $\Delta \ell _2 = 100$ nm, and $\ell _2=800-850$ nm with $\Delta \ell _2 = 10$ nm. As will be seen below (Fig. 3), the reason for considering small values in $\Delta \ell _2$, for the first range (small values of $\ell _2$) and the third range (large values of $\ell _2$), arises from the need to observe in more detail the zones in which the value of $\ell _2$ strongly influences the stability of the inscribed magnetic configurations.

### Simulation software and material parameters

To study and understand the stability of the magnetic configurations and the magnetization reversal processes, we have made use of Mumax3^56^ to numerically solve the Landau–Lifshitz–Gilbert equation given by.

2 $$\begin{aligned} \frac{d \mathbf {m}}{dt} = -\frac{\gamma _0}{1+\alpha ^2}\left[ \mathbf {m} \times \mathbf {H}_{eff} + \alpha \mathbf {m} \times (\mathbf {m} \times \mathbf {H}_{eff}) \right] \end{aligned}$$

where $\mathbf {m}(r,t)$ is the normalized magnetization vector $\mathbf {m}(r,t)=\mathbf {M}(r,t)/M_s$, with $M_s$ as the saturation magnetization, $\gamma _0$ is the gyromagnetic ratio and $\alpha$ is the Gilbert damping constant. The equation describes both the precession and relaxation motion of the magnetization in an effective field $\mathbf {H_{eff}}$. This effective field originates from the system interactions and it is obtained through the functional derivative $\mathbf {H_{eff}} = -\frac{1}{\mu _0 M_s}\frac{\partial \varepsilon \left[ \mathbf {m} \right] }{\partial \mathbf {m}}$ of the energy density functional (see Eq. 3), where $\mu _0$ is a magnetic constant. This functional is originated by the internal interactions $E_{int}$ of the system given by the exchange energy $E_{exc}$ and dipolar $E_{dip}$ and the external ones as the Zeeman energy $E_{Zeeman}$.

3 $$\begin{aligned} E \left[ \mathbf {m} \right]&= E_{exc}+E_{dip}+E_{Zeeman} = \int _V \varepsilon \left[ \mathbf {m} \right] dV \nonumber \\&=\int _V \left( A \sum _{i=1}^3 (\nabla m_i )^2-\frac{\mu _0}{2} \mathbf {H_{dip}}\cdot \mathbf {M}-\mu _0 \mathbf {H}_{ext}\cdot \mathbf {M} \right) dV \end{aligned}$$

For the simulations, we consider permalloy as the material with its properties represented by the following parameters: saturation magnetization of $M_s=800 \times 10^3$ A/m and stiffness constant of $A=13 \times 10^{-12}$ J/m^51^. We have used cell sizes of $2 \times 2 \times 2$ nm$^3$. The damping constant is chosen to a fix value of $\alpha =0.5$ mainly due to get numerical results is shorter time. However, we conducted test calculations with $\alpha =0.1$, 0.05, and 0.01 noticing only slight differences in the coercivity fields (as indicated below) but without changes in the magnetic configurations which is the main point here.

One of the main objectives of the present work is to evaluate the stability of the inscribed magnetic configurations, analyzing possible spontaneous reversals of the magnetization. In solving the LLG equation in time, starting from some initially inscribed configurations of the magnetization, we have applied a cutoff criterion on the torque given by:

4 $$\begin{aligned} \frac{\tau _{max}}{\gamma _0} = \frac{1}{\gamma _0} \text {max} \left( \frac{d \mathbf {m}_i}{dt} \right) \end{aligned}$$

where $d\mathbf {m}_i/dt$ is given by Eq. (2) and $\mathbf {m}_i$ is the magnetization of the i-th cell. In our case, we have considered the following as the cutoff criteria $\tau _{max}/\gamma _0=10^{-4}$ T.

## Results and discussions

The relative chirality of the modulations plays a negligible role in the equilibrium energy, as shown in Fig. 4 of the SI. Thus, such energy is low, of the order to 0.01 eV for the example in the figure and it remains within this order of magnitude for the configurations studied here. However, for configurations with different orientation between segments, the energy differences are of the order of 80 eV (see Fig. 3 of SI). So, for this system, more than three orders of magnitude separate the energy differences due to modulation interactions from segments interactions.

Next illustrative result is given in Fig. 2 where the 4 independent magnetic configurations $C_1$, $C_2$, $C_3$ and $C_4$, left after neglecting the interactions between modulations are shown. $C_1$: the three segments present parallel magnetization; $C_2$: the shorter external segment presents opposite magnetization with respect to the other two segments; $C_3$: the longer external segment presents opposite magnetization with respect to the other two segments; $C_4$: the internal segment presents magnetization opposed to the other two segments. The equilibrium energy of each configuration $E_1$ through $E_4$ is given underneath: it increases in the same order of previous presentation. However, the difference between $C_2$ and $C_3$ is of only 2 eV while the other differences are almost a hundred eV. This is directly related to the number of domain walls pinned within the modulations. A more complete picture is given in Fig. 5 in the SI.

How stable are these configurations with respect to the position of the modulations? Namely, if the magnetization orientation defining each configuration is inscribed in the device, will it remain so or will it spontaneously decay to a lower energy configuration? To answer this question, we have prepared a phase diagram in Fig. 3.

The common abscissa axis in Fig. 3 gives the length $\ell _2$ in nm of the central segment; the ordinates give the proportion in which $\ell _1$ and $\ell _3$ are present according to the $\zeta$ parameter defined in the subsection “Geometrical parameter space”. Each pixel here corresponds to an independent micromagnetic simulation as described in methodology.

Panel (a) corresponds to initializations with $C_1$ configuration that is the true ground configuration so it cannot change. However some of the pixels present configurations with higher energies around $\zeta =0$ and $\ell _2 \approx 810$ nm. Panel (b) represents final states reached by systems initiated as $C_2$. Although most of the parameter space represents stability, those configurations with large enough $\ell _2$ combined with moderate to large values of $|\zeta |$ are unstable and decay to $C_1$ as revealed by the color code. It can be noticed that even for some of the borderline configurations remaining as $C_2$ the energy decreases evidencing the onset for the decay conditions. Panel (c) corresponds to initiations with $C_3$ configuration; the analysis here is identical to previous one due to the symmetry $\zeta \rightarrow -\zeta$ conserving $\ell _2$ that takes $C_2$ into $C_3$. Panel (d) corresponds to configurations initiated as $C_4$ that can decay to $C_2$ and $C_3$ for $|\zeta |$ over a minimum determined by $\ell _2$, which also must be large enough. Moreover, if $\ell _2$ is too large the system initiated as $C_4$ can decay directly into a $C_1$ configuration. All of this is conveniently presented by means of color codes and height of the energy column.

The phase diagram presented in Fig. 3 has been built around the parameter space arising from the system defined in Fig. 1 with the geometrical values valid for the prototype defined above. In case a different geometry is needed the same procedure can be applied to find the stability regions of the desired magnetic configuration storing the information that needs to be protected from spontaneous erasure. Diagram (a) shows that any combination of lengths provides stable configurations $C_1$, which is an obvious result. Diagrams (b) and (c) show in the form of columns (green) the variety of combinations of segments lengths that make configurations $C_2$ and $C_3$ stable enough (metastable). Finally, diagram (d) presents as longer columns (blue) the parameter combinations that make configuration $C_4$ also metastable.

**Figure 4:**
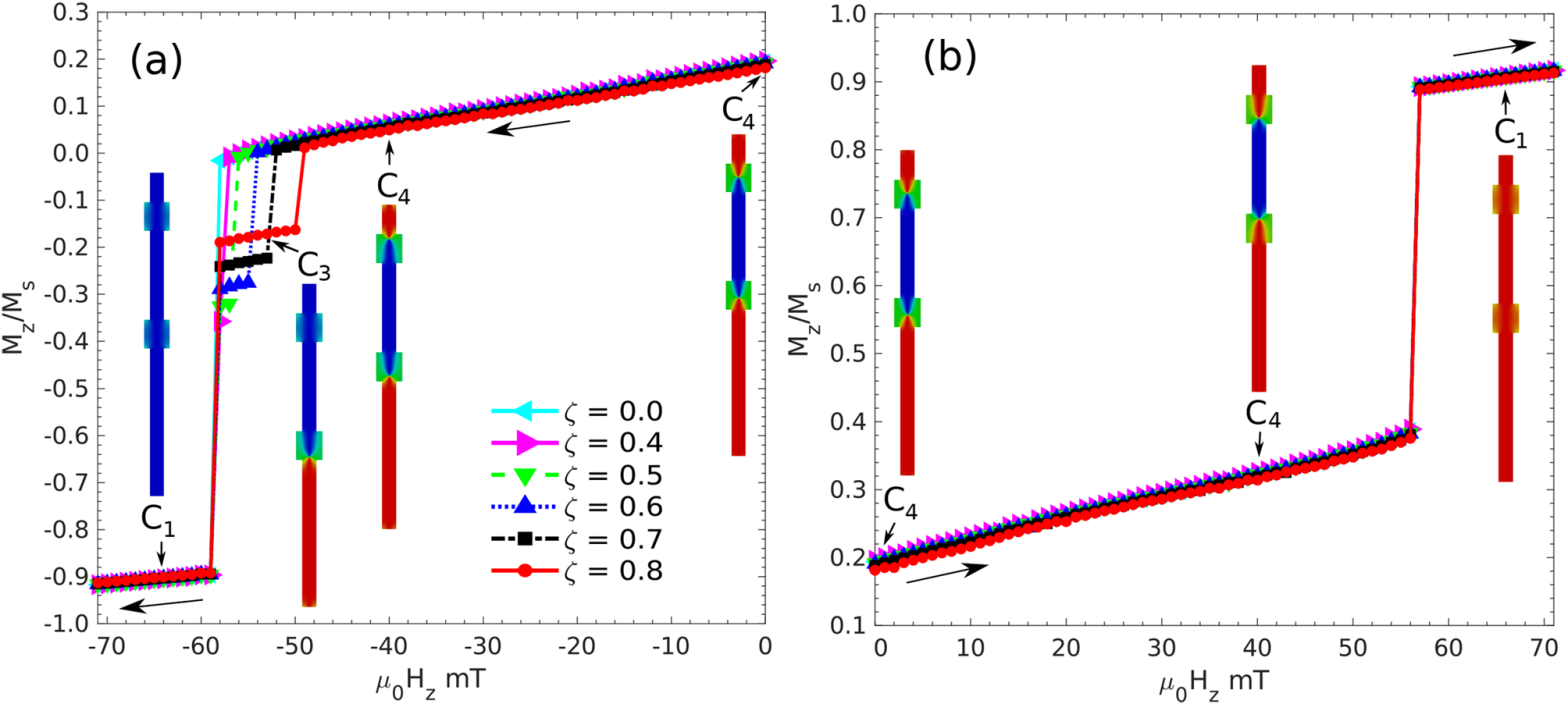
Magnetization curves for an initial $C_4$ configuration and a varying external constant and uniform magnetic field applied in a negative **(a)** and positive **(b)** direction along the z-axis. In this representative example $\ell _2= 300$ nm and different $\zeta$ values are considered as given in the inset. Illustration of the magnetization (using the same color code as before) in the system are given at representative field values for case $\zeta = 0.8$.

But information can also be lost if an external magnetic field comes close to the system and reverses the magnetization in one or more segments. How strong this magnetic fields needs to be? To answer this question for a representative case we present in Fig. 4 the exposition of $C_4$ to an external magnetic field along the *z* direction since this would be the most effective way to reverse the magnetization in the segments thus altering the stored information. Panel (a) presents the case for a constant and uniform magnetic field beginning at zero and increasing its magnitude in the negative *z* direction, while panel (b) does the same but along the positive *z* direction opposite to the magnetization of segment $S_2$. Let us consider the latter first due to its simplicity: the magnetization grows slightly due mainly to polarization within the modulations; actually, BPs tend to get closer to each other. When the magnetic field reaches a value close to $56$ mT segment $S_2$ reverses its magnetization and the system switches to a $C_1$ configuration in an irreversible way.

The increase of the magnetic field towards negative values, namely along the magnetization of the central segment $S_2$ is more complex than previous one. The shorter end segment reverses its magnetization at a field of $-49$ mT switching the configuration to $C_3$ in an irreversible way. If the intensity of the field continues to increase the configuration changes to $C_1$ at a field of about $-59$ mT in an irreversible way.

This way of testing the coercivity fields yields slightly different results depending on the $\alpha$ value used in the simulations. Thus the first coercivity field towards negative external magnetic fields transits from 52 mT to 47 mT as $\alpha$ decreases in a converging way along values 0.5, 0.1, 0.05 and 0.01 for $\zeta =0.7$; the first value is the one reported on the left-hand side of Fig. 4. However, the present work does not attempt to provide precise values which depend on the aim of the device that will be prepared. Here we just show that these configurations are stable enough to store information within normal operating conditions.

These critical fields can be changed a bit upon choosing the lengths of the segments in the desired way. However, the order of magnitude corresponds to the one given in this example, which is more than three orders of magnitude over the average Earth magnetic field. In any case this is a rather high value of a magnetic field for a random contact to a magnetic source. So the system will be robust in most cases where normal magnetic sources are around.

The energy barriers that need to be overcome by the external magnetic fields can be calculated by following the internal magnetic energy along the paths proposed in Fig. 4. This is represented in the Fig. 6 of the SI, for the magnetic field increasing its magnitude along the negative direction (left-hand side of Fig. 4). It is found that the energy barriers that prevent configuration $C_4$ from collapsing to $C_1$ are of the order of 90 eV but can decrease to nearly 60 eV as $\zeta$ increases, where the transition is now from $C_4$ to $C_3$. On the contrary, the energy barrier to jump from $C_3$ to $C_1$ increases with $\zeta$ from zero up to about 25 eV. If the magnetic field is turned off at any point, the system retracts to the last position of minimal magnitude of the magnetic field just above the first energy barrier it finds; the internal energy here turns out to be the same already found when the original configurations were left to equilibrate, namely, attractors are well defined and the situation is reversible after the excess energy is relaxed. This also applies to the vortex domain walls that go back to the equilibrium metastable configurations; eventually different initial states or convergence algorithms need to be used to reach different domain walls configurations^57^, but this is beyond the present scope of this paper.

## Conclusions

A bimodulated cylindrical nanowire can store coded information by means of independent magnetic orientations firmly inscribed on each of its segments. Magnetization on short modulations tends to be chiral, however, the relative vortex orientations do not play a significant role in the equilibrium energy, so the modulations act basically as separators of segments with independent magnetic orientations. However, since the material is homogeneous, such geometric modulations do not represent a difficulty from the deposition point of view.

The configurations are very stable and the energy barriers to be overcome to reverse segments are of the order of some dozens of eV in terms of energy. Thermal excitation at room temperature of the order of few meV is far from providing the necessary energy to erase the stored information. If the same is investigated in terms of external magnetic fields needed to overcome these barriers it is found that dozens of mT are necessary to accomplish this task. Such fields are not randomly available, so a device like this can be very robust.

The system proposed here can be easily extended in several ways. Segments lengths $\ell _1$, $\ell _2$, and $\ell _3$ can be varied to produce a variety of magnetic keys that would properly align the elements of the magnetic lock to let it open. Longer nanowires with many modulations can be proposed to use as firmware to store information in tiny system.

The domain wall inside the modulation separating segments of different magnetic orientation takes the conic form typical of Bloch points (BPs). This is by itself an interesting problem in basic science that we pose here for future developments. Excitation of these systems by means of magnetic pulses can also be used to make use of their internal degrees of freedom. Work is already under way in these two directions.

## Supplementary Information

Supplementary Information.
