# Supplementary material for: Information storage in permalloy modulated magnetic nanowires
Source: Sci Rep. 2021 Oct 21;11:20811. doi: 10.1038/s41598-021-00165-1 (PMC8531287; doi:10.1038/s41598-021-00165-1)
Supplement: Supplementary file 1 — Supplementary Information. [file 41598_2021_165_MOESM1_ESM.pdf]

Information storage in permalloy modulated magnetic nanowires
SUPPORTING INFORMATION

Guidobeth Sáez [a,], Pablo Díaz[a], Eduardo Cisternas[a], Eugenio E. Vogel[a,b], Juan Escrig[b,c]

[a]*Department of Physics, Universidad de La Frontera, Casilla 54-D, Temuco, Chile*
[b]*Center of Nanoscience and Nanotechnology (CEDENNA), 9170124, Santiago, Chile*
[c]*Departamento de Física, Universidad de Santiago de Chile (USACH), Avda. Ecuador 3493, 9170124 Santiago, Chile*

The idea of inscribing magnetic configurations on the segments of the cylindrical bimodulated nanowire stems from results reported for monomodulated nanowires (Ref. [51] in the main text). These results show that the shape anisotropy weakens when a coaxial modulation is added along its main axis. Eventually such system evolved into two independent magnetic segments with the possibility of a domain wall pinned by the modulation.

The proposed geometry of the device is presented in Fig. 1 and subsequent discussion in the main text. We deal with three cylindrical segments S_1, S_2 and S_3 of lengths ℓ_1, ℓ_2 and ℓ_3, respectively, plus two coaxial cylindrical modulations μ_1 and μ_2. Lengths and diameters have been chosen to ensure that the magnetization of the segments is ferromagnetic and along the axis while the magnetization of the isolated modulations can be chiral in any of the two possible rotational degrees of freedom. Considering these five elements as magnetically independent we can inscribe a total of $2^5 = 32$ possible magnetic configurations as presented in Fig. 1 of this Supporting Information (SI). The column to the right corresponds to the one on the left with all magnetization contributions reversed as allowed by the symmetry presented by the Hamiltonian with a quadratic dependence on the magnetic moments. This symmetry property and negligible energy differences (to be seen below) allow to restrict to 4 different magnetic configurations that were presented in Fig. 2 of the main text.

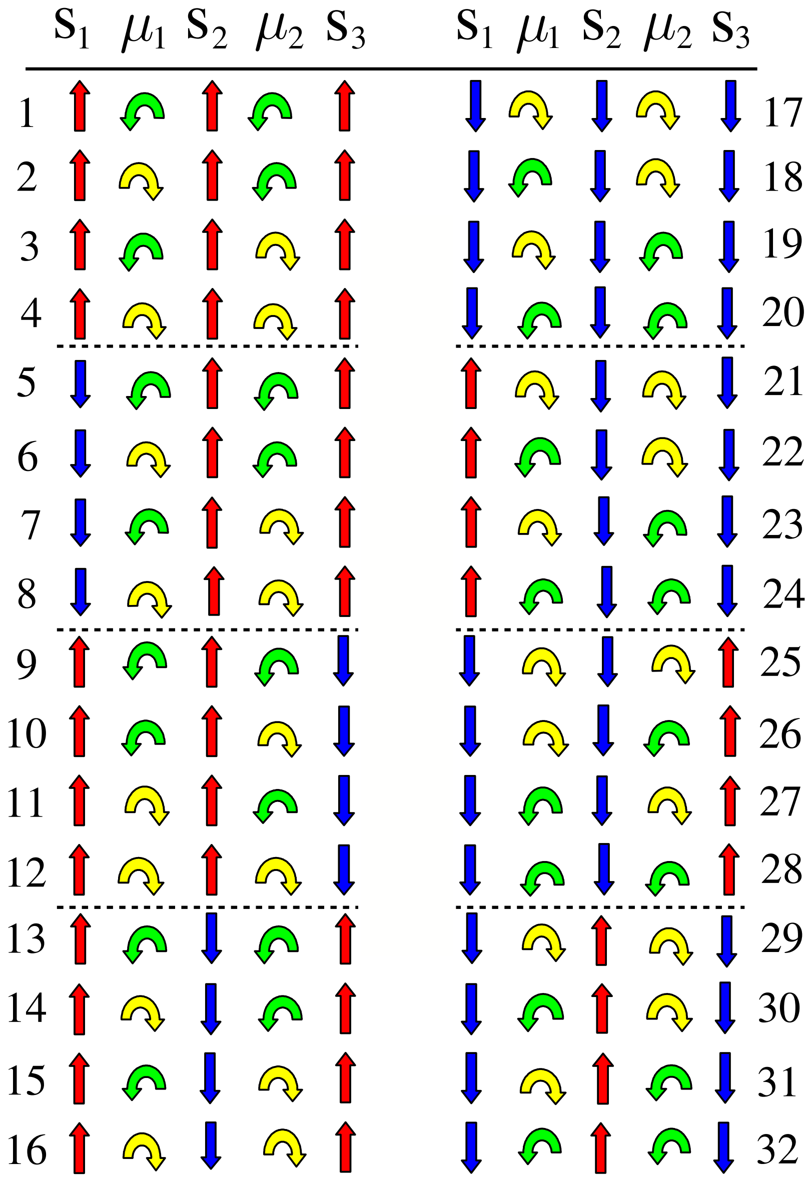

Figure 1: All the 32 possible magnetic configurations with dominant upward magnetization on the left-hand side and downward magnetization on the right-hand side, also varying the chirality within the modulations. Numbers N to the far left and far right are mere labels to identify the configuration in the form G_N.

We assume these configurations can be inscribed in our device by means of external magnetic fields. However, once the external fields are removed, the system relaxes the original energy and searches for the equilibrium configuration that can be different from the originally inscribed. Fig. 2 of SI presents the initial magnetization ($t = 0$ ns) and final (equilibrium) magnetization ($t = 10$ ns) reached by configurations G_9, G_{13}, and G_{14}. These 3 configurations were chosen so G_{13} is an intermediate configuration differing from G_9 in the orientation of one segment, while differing from G_{14} in the orientation of the relative chirality. As it can be seen, G_9 evolves to just a narrowing of the core in the upper modulation while the original straight wall inside the lower modulation evolves into a Bloch point (BP); chirality prevails in a less abrupt way than originally inscribed. In the case of G_{13} both domain walls develop into BPs and both chiralities present slight changes but the relative parallel polarization is preserved. In the case of G_{14} the situation is similar to previous one but here the antiparallel polarization of the chiralities present a slight rotation evidencing that relative orientation is not determinant for the final equilibrium configuration.

Fig. 3 of SI presents the evolution of the equilibration energy for configurations G_9 and G_{13}. The main panel illustrates the fast convergence to the final energy, while the inset gives a better appreciation of the final convergence to an energy difference just under a hundred eV between these two configurations; this can be attributed mainly to the change in the polarity of the two lower segments. The inset also allows to estimate to the equilibration time in about 1.5 ns. As discussed in the main text, simulations using different damping coefficient (α) in the LLG equation can require longer simulation times but only slight changes in coercivities are found.

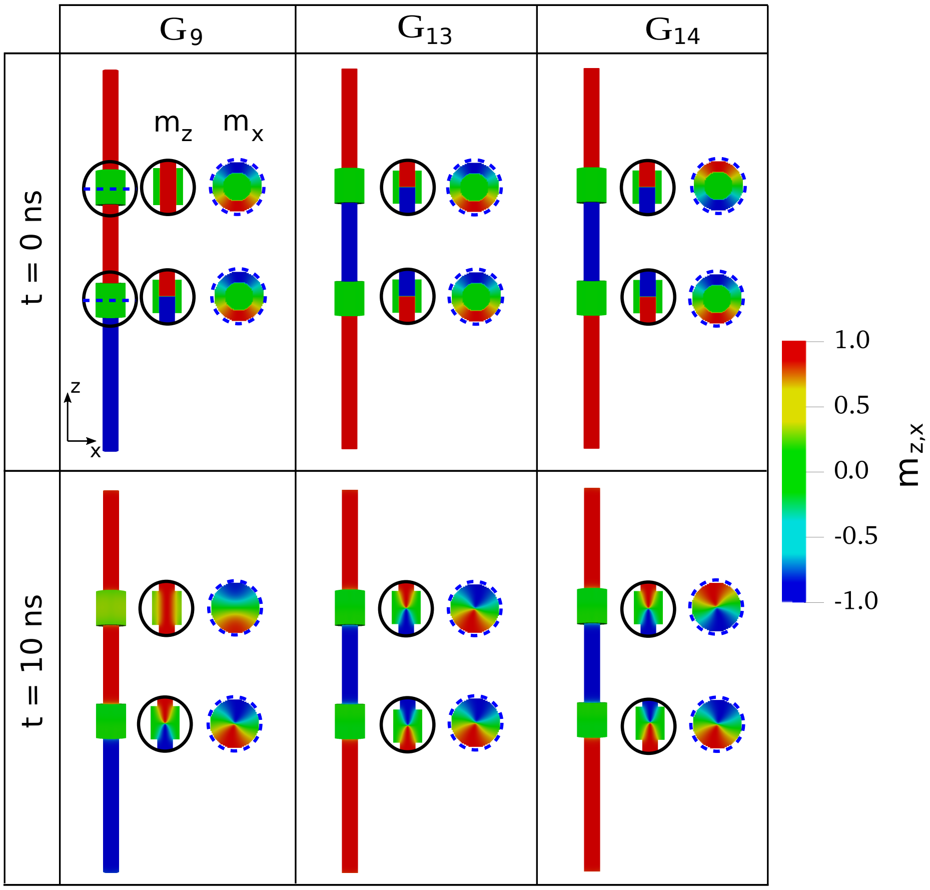

Figure 2: Initial (top) and equilibration or final (bottom) configurations for G_9, G_{13} and G_{14}, for lengths ℓ_1 = 337.5 nm, ℓ_2 = 225 nm, and ℓ_3 = 337.5 nm. Color code for the magnetization components m_z (axial) or m_x (transverse) is given to the right. Continuous circles correspond to a cut showing the internal z components of the magnetization of the as seen portion of the system, while discontinuous circles correspond to transverse cross sections showing the xy field at the center of the modulation.

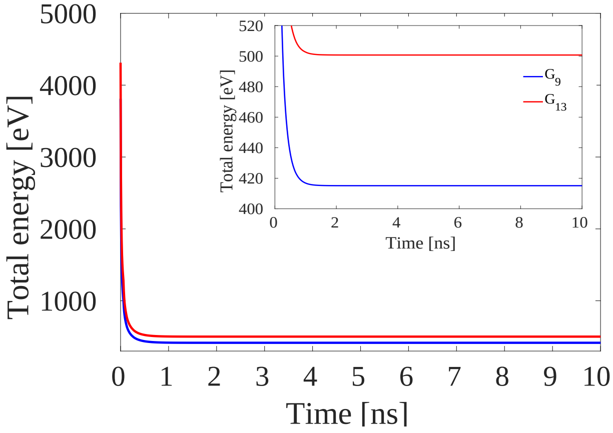

Figure 3: Fast time evolution of the equilibrium energy for the G_9 and G_{13} configurations. The inset zooms on the energy axis showing a difference of over 80 eV.

Fig. 4 of SI does a comparison similar to previous one but now between configurations G_{13} and G_{14} which differ only in the relative direction of the originally inscribed chiralities. The energy difference is less than 0.01 eV. So, we can safely neglect chirality orientations as their energy contributions are way under the intersegment energies. The convergence is fast but equilibrium at this scale is reached at about 4 ns for $\alpha = 0.5$.

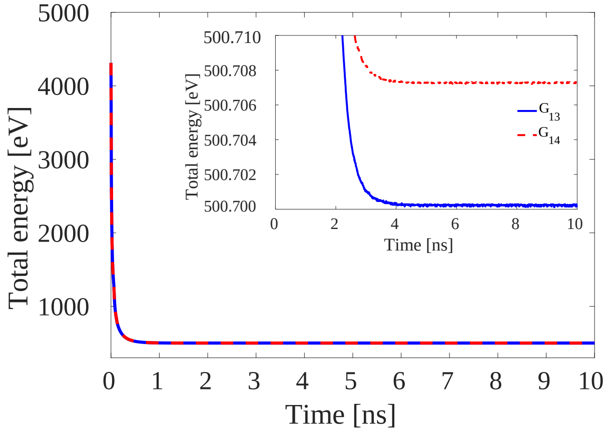

Figure 4: Fast time evolution of the energy for the G_{13} and G_{14} configurations. The inset zooms on the energy axis showing a difference of under 0.01 eV.

Fig. 5 of SI presents the equilibration (or final) energies for the 16 configurations on the left column of Fig. 1 of SI for the geometries defined in the

Table 1 of this SI. These configurations are presented in groups I, II, III and IV according to the orientation of the magnetization in the segments. If we add this condition to the symmetry generated by the quadratic Hamiltonian, then we can reduce the number of configurations from 32 to 4, where we have chosen the configurations G_1, G_5, G_9 y G_{13} presented in this supplementary material as main candidates of study, being renamed as the configurations C_1, C_2, C_3 and C_4, presented in Fig. 2 of the main text. Configurations C_2 and C_3 only differ from each other depending on whether S_2 aligns its magnetization with the shorter or longer external segment. These results were all obtained in the way shown by Figs 3 and 4 of this SI, with 10 ns for equilibration.

Panel	ζ	ℓ_1 nm	ℓ_2 nm	ℓ_3 nm
(a)	0.00	337.5	225	337.5
(b)	0.25	290	225	385
(c)	0.50	225	225	450
(d)	0.75	135	225	540

Table 1: Values of the geometrical parameters, ordered according to the aspect ratio ζ giving the lengths of the segments ℓ_1, ℓ_2, and ℓ_3, keeping $\ell_2 = 225$ nm fixed. First column identifies the panel of configurations in Fig. 5 of SI, and the parameter ζ interrelating ℓ_1 and ℓ_3 is defined in the main text.

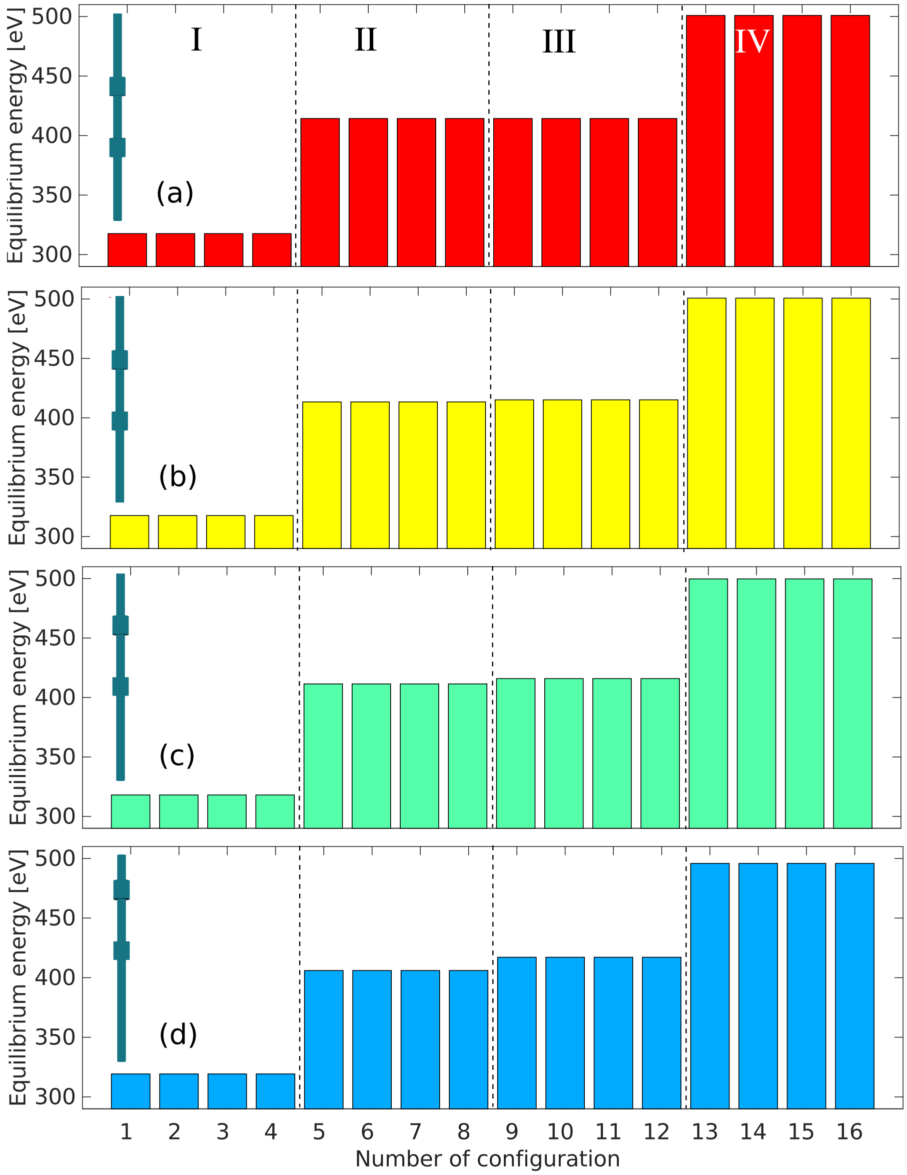

Figure 5: Values of the equilibrium energies, for the 16 configurations G_N listed on the left-hand side of Fig. 1 of this SI. The values of the geometrical parameters are given in the Table of this SI; a graphical representation of the different segment length is given on the left-hand side of each panel.

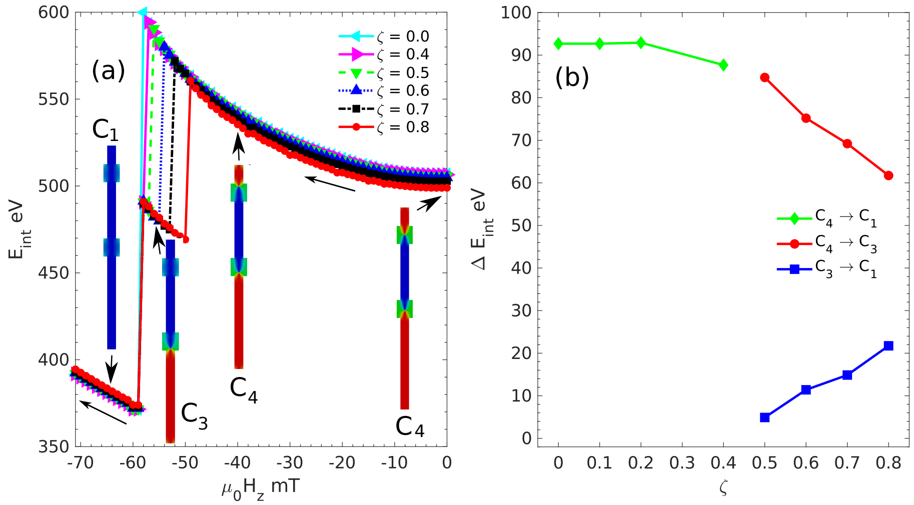

Figure 6: (a) Dependence of the internal energy of the system on the z component of the external magnetic field; the negative magnetic field increases its magnitude from right to left as indicated by the arrows. (b) The energy barriers required to move from one magnetic configuration to next one are plotted for the transitions illustrated in the left-hand side of the figure.

The magnetization curves show that there is a transition between the magnetic configurations, as shown in Figure 4 of the main text, where depending on the ζ value one or two Barkhausen jumps can occur. They originate in the transition from a high energy configuration to a lower energy and more stable configuration. In the present case, this can occur either from C_4 directly to C_1 or from C_4 to C_1 via C_3. Figure (6.a) shows the internal energy curves E_{int} of the system beginning at zero external magnetic field (far right) and increasing due to the external negative magnetic field up to a point a reversal occurs: this represents the energy barrier for this transition. As it can be seen, for $0 \leq \zeta \leq 0.4$ a direct jump from C_4 to C_1 is obtained. For $0.5 \leq \zeta \leq 0.8$ the transition occurs first to C_3 and then with this first energy barrier decreasing with ζ; if the magnetic field continues to increase in magnitude a second energy barrier is overcome . Fig. 6 (b) yields results for the values of these energy barriers for the configurations proposed in this exercise.
